# URAT1 is expressed in cardiomyocytes and dotinurad attenuates the development of diet-induced metabolic heart disease

**DOI:** 10.1016/j.isci.2023.107730

**Published:** 2023-08-25

**Authors:** Yoshiro Tanaka, Tomohisa Nagoshi, Hirotake Takahashi, Yuhei Oi, Rei Yasutake, Akira Yoshii, Haruka Kimura, Yusuke Kashiwagi, Toshikazu D. Tanaka, Masayuki Shimoda, Michihiro Yoshimura

**Affiliations:** 1 Division of Cardiology, Department of Internal Medicine, The Jikei University School of Medicine, 3-25-8, Nishi-Shimbashi, Minato-ku, Tokyo 105-8461, Japan; 2 Department of Pathology, The Jikei University School of Medicine, 3-25-8, Nishi-Shimbashi, Minato-ku, Tokyo 105-8461, Japan

**Keywords:** Cell biology, Pathophysiology, Physiology

## Abstract

We recently reported that the selective inhibition of urate transporter-1 (URAT1), which is primarily expressed in the kidneys, ameliorates insulin resistance by attenuating hepatic steatosis and improving brown adipose tissue function in diet-induced obesity. In this study, we evaluated the effects of dotinurad, a URAT1-selective inhibitor, on the hearts of high-fat diet (HFD)-fed obese mice for 16–20 weeks and on neonatal rat cardiomyocytes (NRCMs) exposed to palmitic acid. Outside the kidneys, URAT1 was also expressed in cardiomyocytes and indeed worked as a uric acid transporter. Dotinurad substantially attenuated HFD-induced cardiac fibrosis, inflammatory responses, and cardiac dysfunction. Intriguingly, among various factors related to the pathophysiology of diet-induced obesity, palmitic acid significantly increased URAT1 expression in NRCMs and subsequently induced apoptosis, oxidative stress, and inflammatory responses via MAPK pathway, all of which were reduced by dotinurad. These results indicate that URAT1 is a potential therapeutic target for metabolic heart disease.

## Highlights

• URAT1 is expressed in the murine heart tissue and cardiomyocytes
• URAT1-selective inhibitor attenuated diet-induced myocardial injury
• PA induced the upregulation of URAT1 in cardiomyocytes
• URAT1-selective inhibitor treatment attenuated PA-induced cardiomyocyte injury

## Graphical abstract

**Figure:**
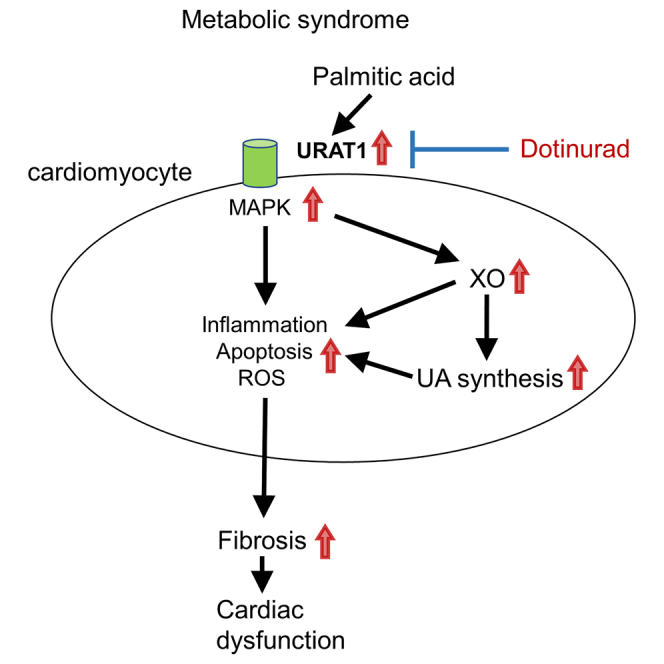

## Introduction

Uric acid (UA) is synthesized via hypoxanthine and xanthine through the purine synthesis pathway by the activation of xanthine oxidase (XO), and is mainly excreted from the kidney into the urine.^1^^,^^2^ Hyperuricemia is classified into the overproduction type and the underexcretion type based on the amount of renal UA excretion.^3^ Renal UA reabsorption is mainly mediated by two UA transporters expressed in proximal convoluted tubules: urate transporter-1 (URAT1) and voltage-driven urate efflux transporter (URATv1).^3^ Other than the kidney, URAT1 is also detected in vascular smooth muscle cells,^4^ vascular endothelial cells,^5^ hepatocytes,^6^^,^^7^ and adipocytes,^7^^,^^8^ and extracellular UA is transported into these cells via URAT1.

High UA levels are often associated with cardiovascular diseases,^2^^,^^9^ and hyperuricemia predicts high morbidity and mortality in patients with cardiovascular diseases.^10^ Patients with hyperuricemia are predisposed to metabolic syndrome, a pathological condition that involves insulin resistance, chronic inflammation, and reactive oxygen species (ROS) synthesis.^11^^,^^12^ The impacts of URAT1 are amplified in metabolic syndrome via persistent exposure to hyperinsulinemia in association with insulin resistance, which results in an increase in UA reabsorption.^13^ Conversely, the enhanced activity of URAT1 induces insulin resistance in metabolic syndrome, leading to a vicious cycle.^7^ The increased UA uptake into cells functions as a pro-oxidant, which results in insulin resistance, ROS synthesis, and inflammation in adipocytes^14^ and HepG2 cells (hepatocyte lineage).^6^ Accordingly, we recently reported that URAT1 is activated in diet-induced obesity (a typical model of metabolic syndrome), and that the activation of URAT1 induces ROS and inflammation in the liver and adipose tissues, leading to the further exacerbation of systemic insulin resistance.^7^ These results suggest that the amplified actions of URAT1 in metabolic syndrome induce cellular injuries in various tissues, although the role and functional significance of URAT1 in myocardial injury associated with metabolic syndrome remain unknown.

Dotinurad, a selective UA reabsorption inhibitor, that selectively inhibits URAT1, has recently been developed as a potent uricosuric agent^7^^,^^15^ and is widely used in clinical practice in Japan for the treatment of patients with hyperuricemia. We recently demonstrated that dotinurad ameliorates insulin resistance by attenuating hepatic steatosis and promoting rebrowning of lipid-rich brown adipose tissue in a mouse model of diet-induced obesity.^7^ We herein hypothesized that dotinurad also attenuates the detrimental impacts of metabolic heart disease in diet-induced obesity. To better understand the role and functional significance of URAT1 inhibition in the pathophysiology of metabolic heart disease, we investigated whether URAT1 is expressed in the heart and—if so—URAT1-selective inhibition directly ameliorates myocardial injury associated with metabolic syndrome.

## Results

### URAT1 is expressed in the murine heart

**Figure 1:**
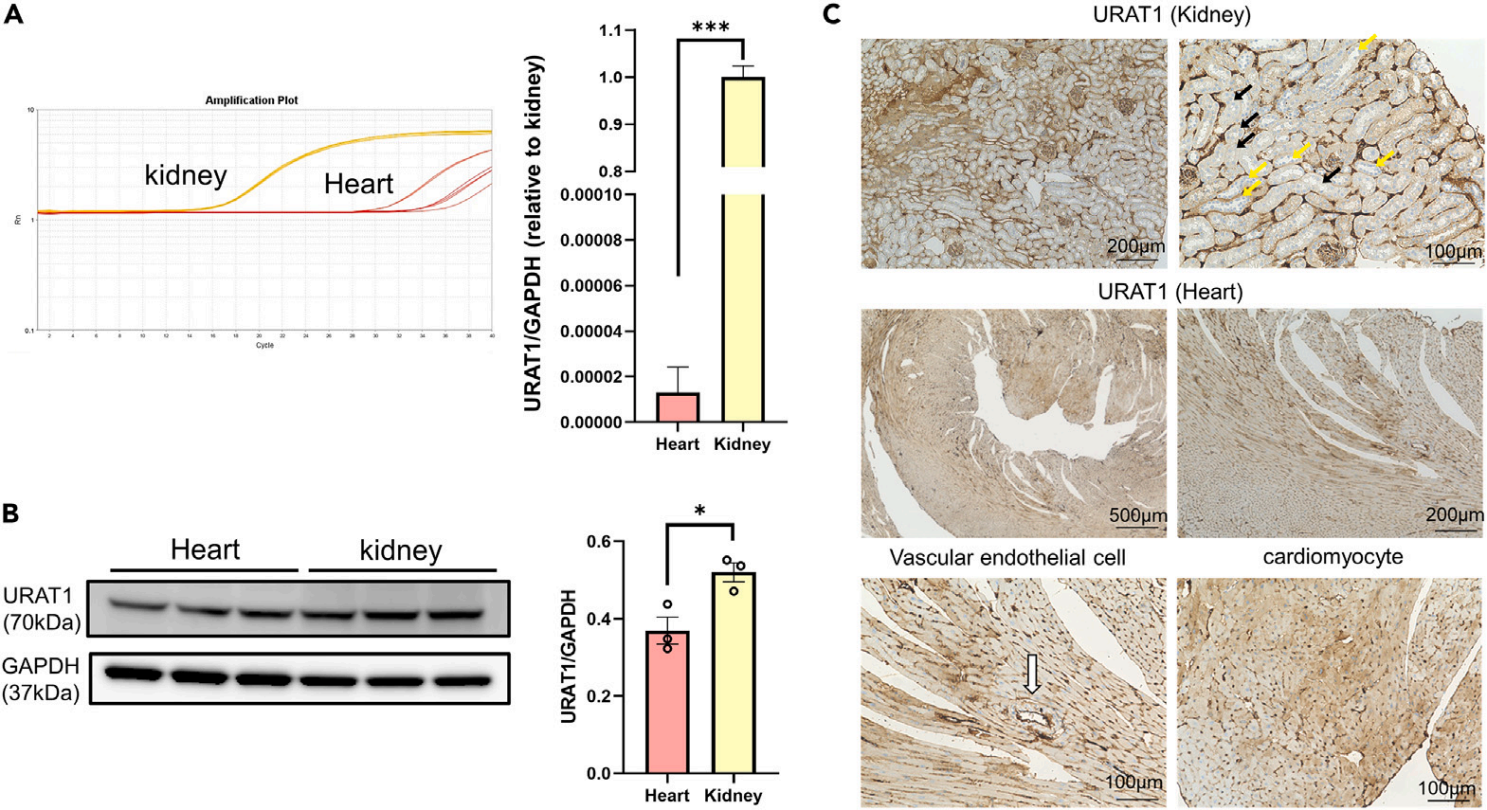
URAT1 is expressed in the murine heart (A) URAT1 mRNA expression in hearts and kidneys obtained from NFD mice, as assessed by qRT-PCR (n = 3 each). (B) The protein expression of URAT1 in hearts and kidneys obtained from NFD mice (n = 3 each). (C) Immunohistochemical analyses of URAT1 expression in kidney cortical sections (upper panels) and hearts (middle panels) in NFD mice. URAT1-positive cells in cardiac vascular endothelial cells (white arrow) and cardiomyocytes are shown in the lower panels. The statistical analysis was performed using a two-tailed Student’s *t* test. Data represent the mean ± SEM. ∗∗∗p < 0.001 and ∗p < 0.05 between the indicated groups. Black arrows indicate proximal convoluted tubules, and yellow arrows indicate distal convoluted tubules.

To determine whether URAT1 is expressed in heart tissue, we first evaluated the expression of URAT1 in the heart and kidney of normal fat diet (NFD)-fed mice. As expected, URAT1 mRNA expression in the kidney was high (Figure 1A). Surprisingly, URAT1 mRNA was also detected in the heart, albeit at relatively low levels compared to those in the kidney. In line with the real-time PCR findings, the expression of URAT1 protein was detected in the heart, although the expression was lower than that in the kidney (Figure 1B). To identify the distribution of URAT1 in the heart, immunohistochemical analyses were performed using kidney and heart tissues obtained from NFD-fed mice (Figure 1C). As expected, immunoreactivity for URAT1 was strong in the proximal convoluted tubules, and URAT1 was also stained in the glomerulus, which is composed of a number of vascular endothelial cells, compared to the distal convoluted tubules; these findings are consistent with the well-known fact that URAT1 is highly expressed in the proximal convoluted tubules and vascular endothelial cells. Under the same protocol using the same anti-URAT1 antibody, URAT1 was successfully stained in the left ventricles, especially in vascular endothelial cells and cardiomyocytes, although the expression was heterogeneous and varied among cells (Figure 1C). These results indicate that URAT1 is substantially expressed in the murine heart.

### URAT1-selective inhibitor treatment attenuates high-fat diet-induced cardiac fibrosis and inflammation

**Figure 2:**
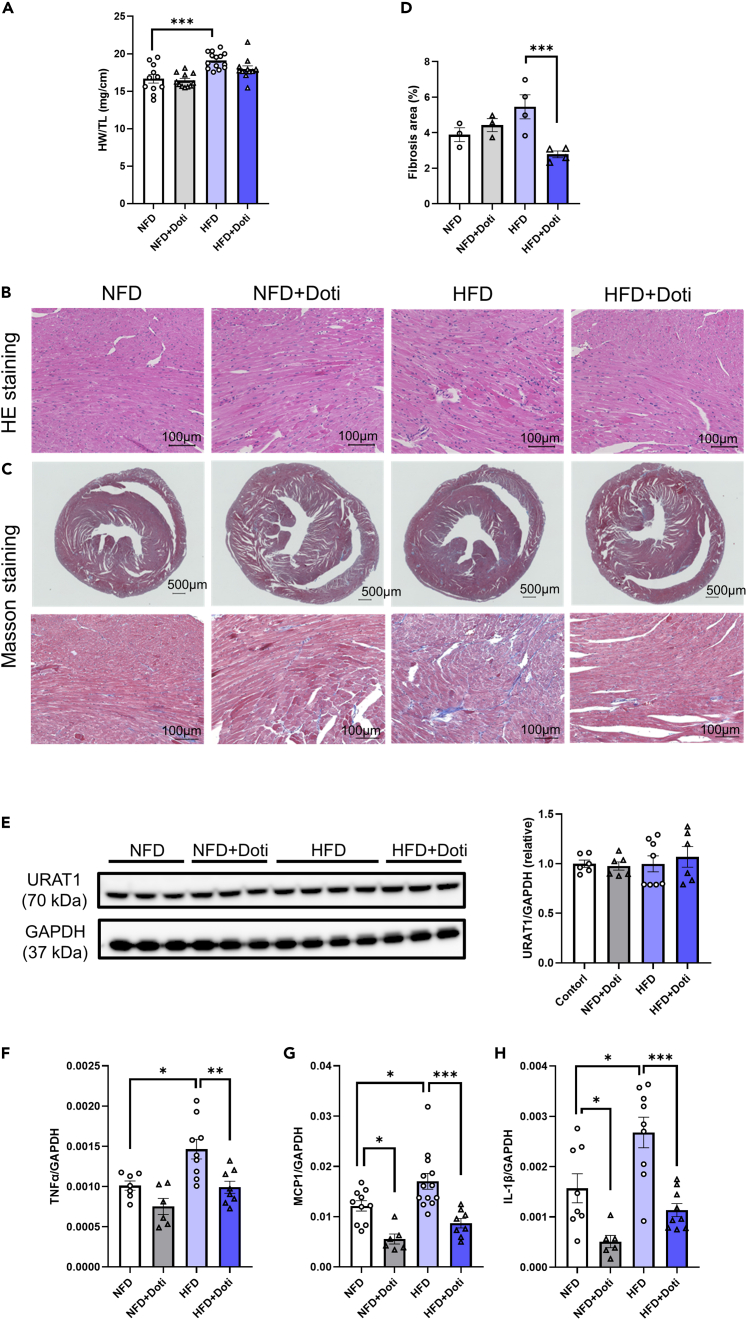
URAT1-selective inhibitor treatment reduces cardiac fibrosis and inflammatory cytokines in HFD-fed mice (A) The heart weight at 4 weeks after treatment with or without dotinurad (NFD, n = 11; NFD+Doti, n = 11; HFD, n = 14; HFD+Doti, n = 12). (B) Representative hematoxylin-eosin staining of cardiac tissues. (C) Representative micrographs of histochemical staining of connective tissues using Masson’s trichrome. (D) The area of fibrosis assessed by Masson’s trichrome staining (NFD, n = 3; NFD+Doti, n = 3; HFD, n = 4; HFD+Doti, n = 4). (E–H) URAT1 protein expression in NFD and HFD mice with or without dotinurad treatment (NFD, n = 6; NFD+Doti, n = 6; HFD, n = 8; HFD+Doti, n = 6). The relative mRNA expression levels of TNF-α (F), MCP1 (G), and IL-1β (H) in heart tissue ([TNFα] NFD, n = 7; NFD+Doti, n = 6; HFD, n = 9; HFD+Doti, n = 8; [MCP1] NFD, n = 10; NFD+Doti, n = 6; HFD, n = 13; HFD+Doti, n = 8; [IL-1β] NFD, n = 8; NFD+Doti, n = 6; HFD, n = 9; HFD+Doti, n = 9). The statistical analysis was performed using one-way ANOVA followed by Tukey’s post hoc test. Data represent the mean ± SEM. ∗∗∗p < 0.001, ∗∗p < 0.01, and ∗p < 0.05 between the indicated groups. HE, hematoxylin-eosin, HW, heart weight; TL, tail length; NFD, normal fat diet; HFD, high-fat diet; Doti, dotinurad.

To evaluate the pathophysiological role of cardiac URAT1 in high-fat diet (HFD)-induced myocardial injury, we next examined the effects of dotinurad, a URAT1-selective inhibitor, on heart tissue in HFD-fed mice. The heart weight in HFD-fed mice was significantly increased in comparison to that in NFD-fed mice (Figure 2A), but treatment with dotinurad did not significantly decrease it (HFD vs. HFD+dotinurad, p = 0.12). Histological studies showed myofiber disorganization (Figure 2B) and cardiac fibrosis (Figures 2C and 2D) in HFD-fed mice compared to NFD-fed mice, which were substantially ameliorated by dotinurad treatment. We next examined the effects of diet conditions and dotinurad treatment on URAT1 expression in heart tissue, and we found that URAT1 expression was comparable between the groups (Figure 2E).

HFD feeding for 16–18 weeks leads to hyperglycemia, hyperuricemia, and hyperinsulinemia, as we previously demonstrated.^7^ These pathological conditions of metabolic syndrome induce inflammation in the cardiac tissue; increase in tumor necrosis factor alpha (TNF-α), monocyte chemotactic protein-1 (MCP1), and interleukin 1β (IL-1β), all of which are M1 macrophage markers and important pathological characteristics in HFD-induced myocardial injury.^16^^,^^17^ We found that HFD feeding significantly increased the gene expression of TNF-α, MCP1, and IL-1β in the heart, which was substantially suppressed by dotinurad treatment (Figures 2F–2H). These results indicate that a URAT1-selective inhibitor significantly ameliorates HFD-induced cardiac fibrosis and inflammation.

The cardiac expression of CD68, a valuable macrophage marker in the histochemical analysis of inflamed tissues,^16^ showed a trend toward an increase in HFD-fed mice in comparison to NFD-fed mice (p = 0.08), although dotinurad did not reduce it (Figure S1A). A recent study showed that serum UA induces an NLRP3 inflammasome-dependent mechanism in the heart.^18^ Although HFD induced the cardiac expression of NLRP3, dotinurad did not affect the expression of NLRP3 (Figure S1B).

### Effects of URAT1-selective inhibitor treatment on HFD-induced cardiac dysfunction

**Figure 3:**
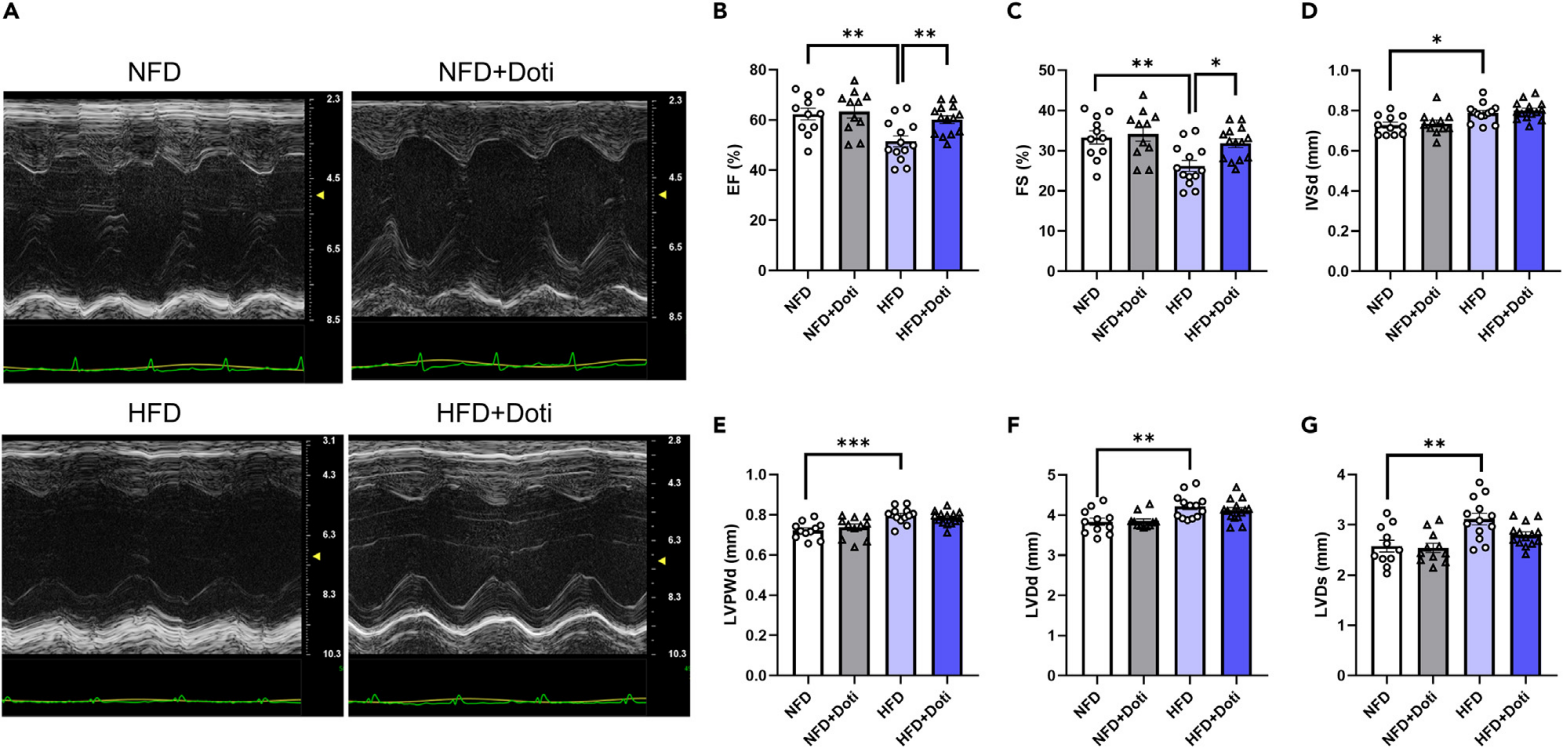
URAT1-selective inhibitor treatment ameliorates HFD-induced cardiac dysfunction as assessed by echocardiography (A) Representative M-mode echocardiograms obtained from NFD or HFD mice with or without dotinurad. (B–G) The data of the indicated echocardiographic parameters are shown (NFD, n = 11; NFD+Doti, n = 11; HFD, n = 13; HFD+Doti, n = 14). The statistical analysis was performed using one-way ANOVA followed by Tukey’s post hoc test. Data represent the mean ± SEM. ∗∗∗p < 0.001, ∗∗p < 0.01, and ∗p < 0.05 between the indicated groups. Doti, dotinurad; EF, ejection fraction; FS, fractional shortening; HFD, high-fat diet; IVSd, diastolic thickness of intraventricular septum; LVDd, left ventricular end-diastolic dimension; LVDs, left ventricular end systolic dimension; LVPWd, left ventricular posterior wall thickness; NFD, normal fat diet.

To test the functional significance of URAT1 in metabolic heart disease, we next assessed cardiac function by echocardiography at four weeks after the administration of dotinurad. Consistent with a previous study,^19^ the left ventricular ejection fraction (LVEF) and fractional shortening (FS) were significantly impaired in HFD-fed mice in comparison to NFD-fed mice, in which the LVEF and FS were ameliorated by dotinurad treatment (Figures 3A–3C; Table S1 and Videos S1, S2, S3, and S4). The end diastolic thickness of the intraventricular septum, end diastolic thickness of the posterior wall, left ventricular end diastolic dimension, and left ventricular end systolic dimension (LVDs) were significantly increased in HFD-fed mice in comparison to NFD-fed mice. Among these parameters, dotinurad tended to decrease the LVDs in HFD-fed mice (HFD vs. HFD+dotinurad, p = 0.06). These results indicate that the selective inhibition of URAT1 significantly improves diet-induced cardiac dysfunction, presumably through the inhibition of cardiac inflammation and fibrosis, as shown in Figure 2.

Video S1. Representative short axis view of echocardiography obtained from NFD mouse, related to Figure 3

Video S2. Representative short axis view of echocardiography obtained from NFD mouse with dotinurad treatment (NFD+Doti), related to Figure 3

Video S3. Representative short axis view of echocardiography obtained from HFD mouse, related to Figure 3

Video S4. Representative short axis view of echocardiography obtained from HFD mouse with dotinurad treatment (HFD+Doti), related to Figure 3

The beneficial *in vivo* effects of dotinurad in terms of ameliorating the cardiac remodeling and function could also be indirectly mediated by the action of dotinurad on other tissues, such as the kidney.^20^ However, at least, plasma Cr levels were comparable between HFD and NFD, and dotinurad treatment did not affect them (Figure S2), consistent with our previous study.^21^

### URAT1 in cardiomyocytes functions as a UA transporter

**Figure 4:**
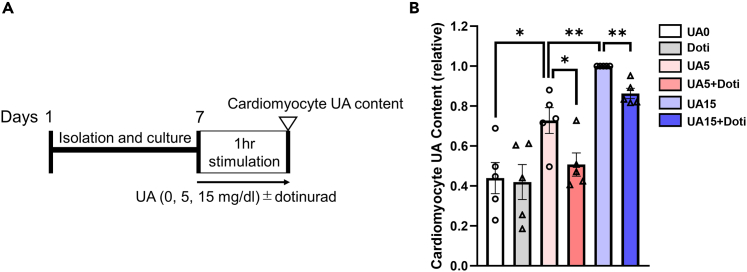
Cardiomyocytes take up UA via URAT1 (A) A schematic diagram of the experimental protocol. (B) The intracellular UA levels in NRCMs exposed to the indicated treatments for 60 min (n = 5 each). The statistical analysis was performed using a two-tailed Student’s *t* test or Mann‒Whitney U test. Data represent the mean ± SEM. ∗∗p < 0.01 and ∗p < 0.05 between the indicated groups. UA, uric acid.

To determine whether extracellular UA is transported into cardiomyocytes through URAT1, we next examined UA uptake in neonatal rat cardiomyocytes (NRCMs) (Figure 4A). When NRCMs were exposed to UA in the medium (5 or 15 mg/dL) for 60 min, the intracellular UA levels were significantly increased in a UA concentration-dependent manner. This was substantially decreased by treatment with dotinurad (Figure 4B). These results demonstrate that URAT1 actually transports UA into cardiomyocytes and that dotinurad inhibits the function of cardiac URAT1.

Previous studies have shown that a high concentration of UA induces the upregulation of URAT1 in tubular epithelial cells^22^ and inflammatory signals in myocytes.^18^ Thus, we next examined the effects of UA on the expression of URAT1 and inflammatory cytokines in NRCMs. However, when NRCMs were exposed to UA (concentration: 5 or 15 mg/dL) for 24 h, high UA did not increase the expression of URAT1 in NRCMs, and dotinurad did not significantly affect the expression of URAT1 in NRCMs (Figure S3A). Likewise, neither high UA nor dotinurad changed the MCP1 or IL-1β levels in the NRCMs (Figures S3B and S3C). These results indicate that UA *per se* does not affect the expression levels of URAT1 and inflammatory cytokines in cardiomyocytes, at least in the present model.

### Palmitic acid enhances the expression of URAT1 in NRCMs

One of the main pathophysiological mechanisms of metabolic syndrome is insulin resistance, which results in hyperinsulinemia, and insulin has been shown to increase the expression of URAT1 in kidney epithelial cells.^13^ Thus, we examined the effects of insulin on the expression of URAT1 in NRCMs. However, high glucose (4.5 g/L) and insulin (100 nM) did not have a significant impact on URAT1 expression in cardiomyocytes (Figure S4).

**Figure 5:**
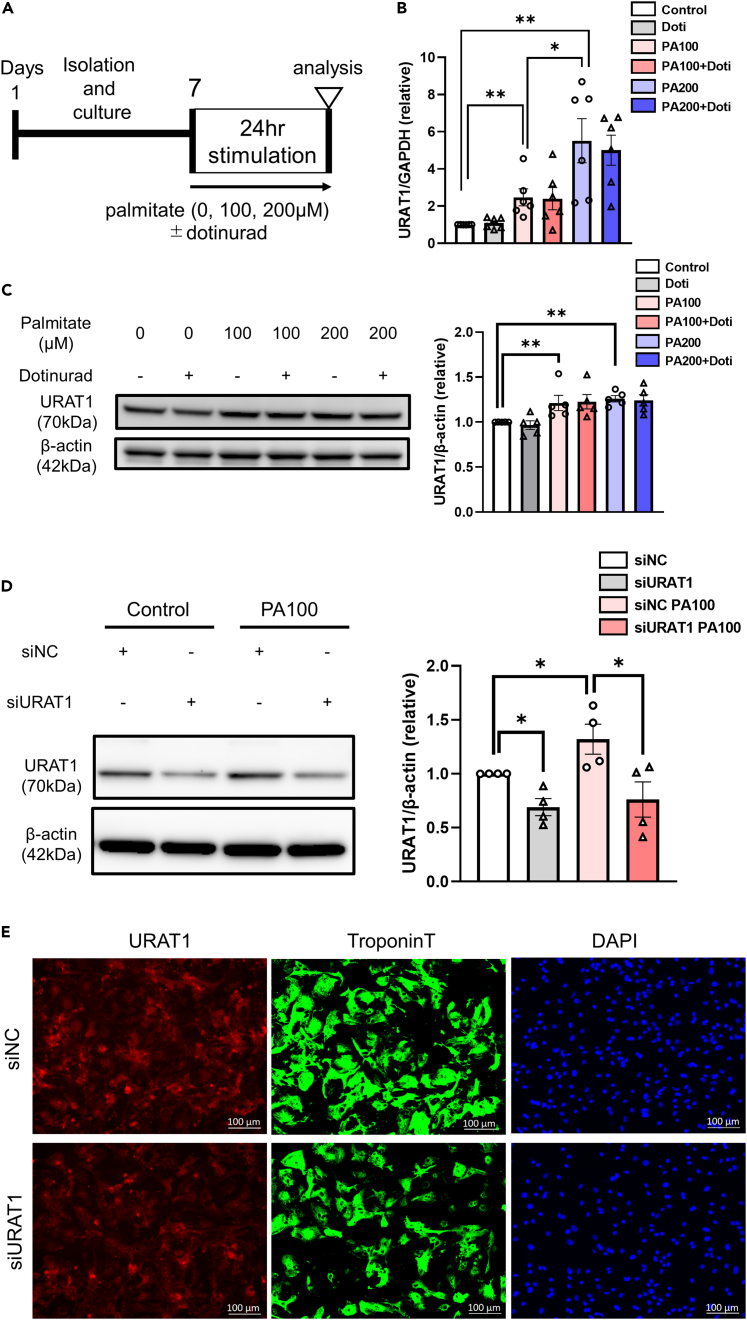
PA induced the expression of URAT1 in NRCMs (A–C) A schematic diagram of the experimental protocol. URAT1 mRNA (n = 6 each) (B) and protein expression (n = 5 each) (C) in NRCMs exposed to the indicated treatments for 24 h. (D) Representative immunoblots and quantitative analyses of URAT1 in NRCMs transfected with URAT1 siRNA (siURAT1) or vehicle control siRNA (siNC) exposed to the indicated treatments for 24 h. (E) Immunofluorescence analyses of URAT1 expression in NRCMs transfected with siURAT1 or siNC. Cells were stained with URAT1 (red), Troponin-T (Green), and DAPI (blue). The statistical analysis was performed using a two-tailed Student’s *t* test or Mann‒Whitney U test in (B–D). Data represent the mean ± SEM. ∗∗p < 0.01 and ∗p < 0.05 between the indicated groups. PA, palmitate; Doti, dotinurad.

Saturated fatty acids, such as palmitic acid (PA), are contained in various types of unhealthy food, and the excess intake of saturated fatty acids leads to obesity and metabolic syndrome.^17^ In fact, we confirmed that the plasma PA concentration in mice fed HFD for 32–34 weeks was significantly increased in comparison to that in mice fed NFD ([NFD] 576 ± 64 μg/mL vs. [HFD] 805 ± 46 μg/mL, p < 0.05); thus, it is reasonable to assume that, among the various dietary ingredients composing HFD, PA has a substantial impact on the development of metabolic heart disease. When NRCMs were exposed to PA (100 or 200 μM) for 24 h (Figure 5A), URAT1 mRNA expression in NRCMs was dramatically upregulated in a PA concentration-dependent manner (Figure 5B). In line with this, PA (100 or 200 μM) significantly increased the protein expression of URAT1 in comparison to PA-free controls (Figures 5C and 5D). As expected, neither the mRNA nor the protein expression of URAT1 was affected by dotinurad.

To confirm the presence of URAT1 in cardiomyocytes and to see the effects of URAT1 on cardiomyocytes more specifically (as we shall see later), we knocked down URAT1 in NRCMs by a small interfering RNA (siRNA) gene silencing system. In line with the immunoblotting data on URAT1 expression (Figures 5C and 5D), immunofluorescence showed that URAT1 was expressed in cardiomyocytes (confirmed by double staining with Troponin T, a specific marker of cardiomyocytes) (Figure 5E), and transfection with URAT1-specific siRNA (siURAT1) significantly reduced its expression in cardiomyocytes compared to that with vehicle control siRNA (siNC) (Figures 5D and 5E). Likewise, we confirmed that the PA-induced increase in URAT1 expression was abrogated by siURAT1 (Figure 5D).

URAT1 mRNA and protein were also detected in neonatal cardiac fibroblasts. However, PA did not affect either the mRNA or protein expression of URAT1 (Figures S5A and S5B). Furthermore, we also found URAT1 protein expression in human umbilical vein endothelial cells (HUVECs), but PA did not affect them in a PA concentration-dependent manner (Figure S5C). These results suggest that PA is a key regulator of the upregulation of URAT1 in cardiomyocytes, and this effect may be a distinctive feature of cardiomyocytes among other cardiac cells.

### URAT1-selective inhibitor treatment reduces PA-induced apoptosis, ROS, and inflammation in NRCMs by inhibiting MAPK signaling

**Figure 6:**
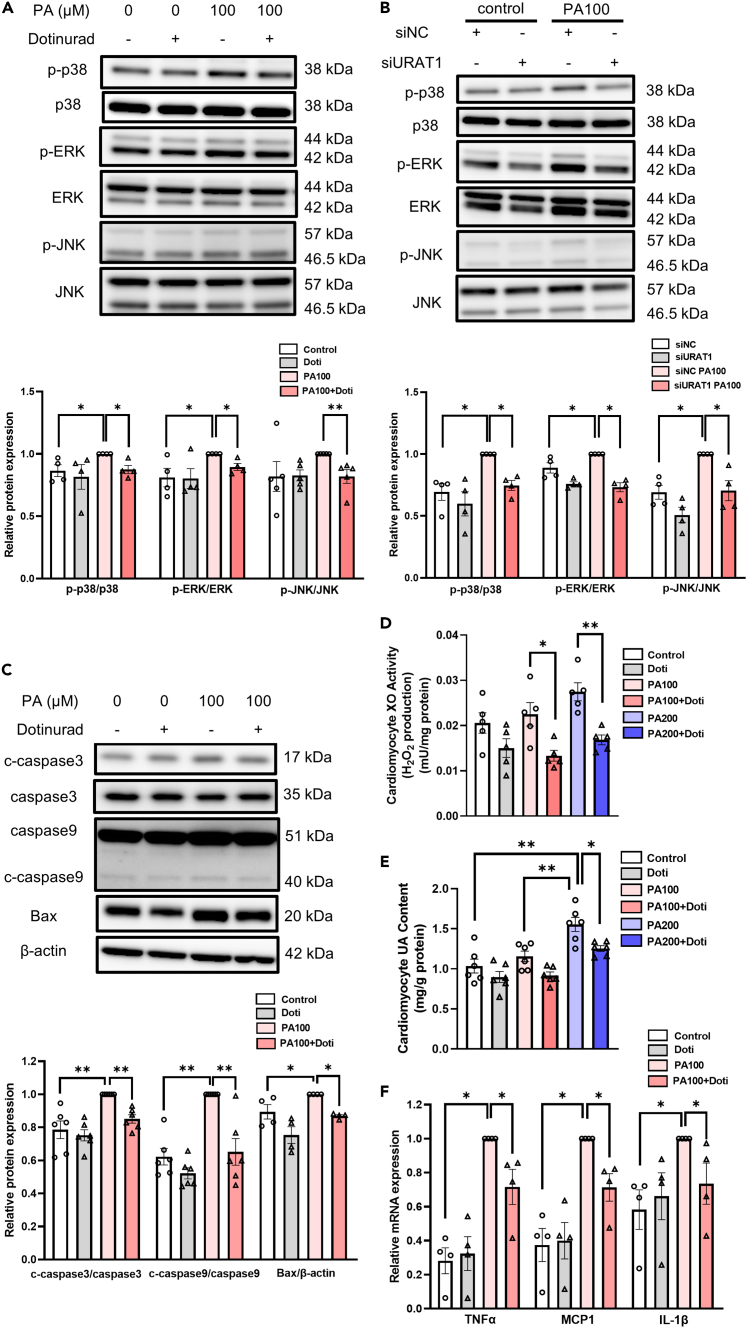
URAT1-selective inhibitor alleviates PA-induced MAPK phosphorylation, apoptosis, ROS, and inflammation in NRCMs (A) Representative immunoblots (upper) and quantitative analyses (lower) of p-p38, p38, *p*-ERK, ERK, *p*-JNK, and JNK from NRCMs exposed to the indicated treatments for 24 h ([p-p38/p38] and [p-ERK/ERK] n = 4 each; [p-JNK/JNK] n = 5 each). (B) Representative immunoblots (upper) and quantitative analyses (lower) of p-p38, p38, *p*-ERK, ERK, *p*-JNK, and JNK from NRCMs transfected with URAT1 siRNA (siURAT1) or vehicle control siRNA (siNC) exposed to the indicated treatments for 24 h (n = 4 each). (C) Representative immunoblots and quantitative analyses of the indicated apoptosis markers from NRCMs exposed to the indicated treatments for 24 h ([c-caspase 3] and [c-caspase 9] n = 6 each; [Bax] n = 4 each). (D) XO activity (H_2_O_2_ production using xanthine as a substrate) in NRCMs exposed to the indicated treatments for 24 h (n = 5 each). (E) The intracellular UA levels in NRCMs exposed to the indicated treatments for 24 h (n = 6 each). (F) The relative mRNA expression of the indicated inflammatory markers in NRCMs exposed to the indicated treatments for 24 h (n = 4 each). The statistical analysis was performed using a Mann‒Whitney U test in (A, B, C, and F) or a one-way ANOVA followed by Tukey’s post hoc test in (D and E). Data represent the mean ± SEM. ∗∗p < 0.01 and ∗p < 0.05 between the indicated groups. ROS, reactive oxygen species; PA, palmitate; Doti, dotinurad; XO, xanthine oxidase; UA, uric acid.

Previous studies have shown that PA-induced apoptosis, ROS, and inflammation are mediated through MAPK pathways in myocytes.^23^^,^^24^ When NRCMs were exposed to PA (100 μM) for 24 h, the phosphorylation of ERK and p-38 was significantly increased, and the phosphorylation of JNK was nonsignificantly (p = 0.13) increased in NRCMs (Figure 6A). Treatment with dotinurad significantly suppressed the PA-induced phosphorylation of p-38, ERK, and JNK in NRCMs. Furthermore, the PA-induced phosphorylation of p-38, ERK, and JNK was significantly reduced in NRCMs transfected with siURAT1 (Figure 6B), which is consistent with the results obtained using dotinurad.

As a result of the phosphorylation of MAPK by treatment with PA, PA induced the protein expression of cleaved caspase-3, cleaved caspase-9, and Bax in NRCMs, and this effect was significantly ameliorated by dotinurad treatment (Figure 6C). In line with this, the PA-induced upregulation of cleaved caspase-9 and Bax, but not cleaved caspase-3 (for reasons that are unclear), was significantly reduced in NRCMs transfected with siURAT1 (Figure S6).

XO is an important source of ROS in the failing heart. The Amplex Red fluorescence assay for XO activity measurement used in the present study detects tissue hydrogen peroxide (H_2_O_2_) production induced by XO activation, namely by measuring XO-derived ROS levels in NRCMs.^1^^,^^25^ When NRCMs were exposed to a high concentration of PA (200 μM) for 24 h, the XO-derived H_2_O_2_ levels (namely, the XO activity) in NRCMs increased compared to the control, although the difference did not reach statistical significance (p = 0.0504). However, dotinurad treatment significantly reduced the XO-derived H_2_O_2_ levels in PA-treated NRCMs (100 or 200 μM) (Figure 6D). These results indicate that PA-induced XO activation and ROS synthesis in NRCMs were ameliorated by treatment with dotinurad. As such, PA (200 μM) substantially reduced cell viability, which was significantly attenuated by dotinurad treatment (Figure S7). We previously reported that XO catalyzes the production of UA in cardiomyocytes.^1^ As expected, PA significantly increased the accumulation of UA in NRCMs in a PA concentration-dependent manner, which was significantly reduced by dotinurad (PA 200 μM) (Figure 6E). Given that the cardiomyocytes were cultured in UA-free medium, these PA-induced observations are thought of as exogenous UA-independent phenomena.

In contrast, PA did not enhance NLRP3 expression in NRCMs, and dotinurad did not affect NLRP3 expression, consistent with the whole heart tissue findings (Figure S1B), suggesting that the NLRP3 inflammasome-dependent inflammatory pathway is not deeply involved in PA-induced myocardial injury, at least in the present experimental model (Figure S8). However, PA (in contrast to UA overload [Figures S3B and S3C]) significantly upregulated inflammatory cytokines in NRCMs, such as TNF-α, MCP1, and IL-1β, which were significantly reduced by dotinurad treatment (Figure 6F). These data suggest that dotinurad ameliorates PA-induced apoptosis, ROS, and inflammatory cytokines by inhibiting URAT1 in cardiomyocytes in an “extracellular” UA-independent mechanism.

## Discussion

In the present study, we proposed and verified the role and functional significance of URAT1 in myocardial injury and cardiac dysfunction observed in HFD-induced obesity. The remarkable findings in the present study are that—outside of the kidney—URAT1 is expressed in cardiomyocytes and functions as an UA transporter. Among various potential factors that regulate the expression and activity of URAT1, we found that PA, which is increased in metabolic syndrome, upregulates URAT1 expression in cardiomyocytes. Accordingly, myocardial URAT1 substantially contributes to the development of diet-induced metabolic heart disease; PA-induced URAT1 upregulation in cardiomyocytes induces apoptosis, ROS synthesis, and the inflammatory response via the MAPK pathway, which results in enhanced cardiac fibrosis, ultimately leading to cardiac dysfunction. Dotinurad, a URAT1-selective inhibitor, remarkably attenuates these detrimental impacts of diet-induced metabolic heart disease and may be a potential therapeutic agent for metabolic heart disease-associated hyperuricemia (Graphical Abstract).

URAT1 is known to be expressed in various types of cells other than kidney cells.^4^^,^^5^^,^^6^^,^^7^^,^^8^ However, previous studies showed that there is no URAT1 mRNA in the adult human heart by northern blotting^26^ or in HL-1 myocytes (an adult murine myocyte cell line).^27^ In contrast, we determined URAT1 expression in the mouse heart by RT-qPCR (Figure 1A), immunoblotting (Figure 1B), and immunohistochemistry (Figure 1C). The evidence for URAT1 expression in the heart was confirmed by an immunofluorescence study (double staining for troponin T, a specific marker of cardiomyocytes, and URAT1) and by URAT1-specific knockdown experiments using siRNA in NRCMs. We also found that URAT1 was expressed in cardiac fibroblasts and HUVECs. What is more remarkable is that URAT1 actually transports UA into cardiomyocytes and that dotinurad, a URAT1-selective inhibitor, directly suppresses the uptake of UA into cardiomyocytes. These results convinced us that the oral administration of dotinurad substantially inhibits cardiac URAT1 both *in vitro* and *in vivo*.

The upregulation of URAT1 protein in cardiomyocytes by treatment with PA is a new observation in the field that was not observed in cardiac fibroblasts and HUVECs. This finding suggests that the impact of PA on the URAT1 expression might differ depending on the cell type. Thus, although the URAT1 expression in cardiomyocytes alone was significantly increased by PA exposure, it might have been attenuated in whole heart tissues (as shown in Figure 2E), which comprise various types of cells, such as fibroblasts and endothelial cells. It can also be argued that the effect of PA on URAT1 upregulation is relatively specific in cardiomyocytes among cardiac cells as a whole.

Previous studies showed that tissue expression and actions of URAT1 in the kidney and adipose tissues are enhanced in an insulin-resistant state, presumably via persistent exposure to hyperinsulinemia, hyperuricemia, and hypercholesterolemia, including high PA (namely, the factors all of which are deeply involved in the pathophysiology of metabolic syndrome and could develop the current model of metabolic heart disease).^7^^,^^13^^,^^28^ Thus, some of the components of metabolic syndrome may play a critical role in the activation of URAT1 in cardiomyocytes. However, high UA *per se* did not induce cardiomyocyte injury in the present study, which is not consistent with previous studies.^18^^,^^29^^,^^30^ One of the reasons for the discrepancy is that H9c2 cells were used in the previous study, and we assume that H9C2 cells, which show quite different cellular characteristics from actual cardiomyocytes, might be more susceptible to high UA than cardiomyocytes. Furthermore, in the present study, high glucose values combined with insulin overload did not affect the expression of URAT1 in cardiomyocytes (Figure S4). Thus, among the various factors involved in the pathophysiology of diet-induced obesity, PA is the only one that induced URAT1-mediated oxidative stress and inflammation in cardiomyocytes, at least in the present study. Based on these findings, combined with the facts that PA is a main component of HFD and the plasma PA concentration was actually increased in the current HFD mice, NRCMs exposed to PA may be a suitable *in vitro* experimental setting reflecting the current *in vivo* HFD-induced mouse model for investigating the role of cardiomyocyte URAT1 in metabolic heart disease. Therefore, in the present study, we focused on PA, one of the major contributing factors to insulin resistance in metabolic disorders,^31^ as a potential positive regulator of cardiomyocyte URAT1 (Figures 5B–5D). Accordingly, when mice are exposed to high PA (e.g., in metabolic syndrome), the action of URAT1 in cardiomyocytes is enhanced, and URAT1-selective inhibition has more potent effects in the HFD mouse heart than in the NFD mouse heart. Taken together, hyperuricemia associated with high PA, such as metabolic syndrome, may lead to the activation of URAT1 in cardiomyocytes, and a URAT1-selective inhibitor has a significant impact on PA-induced myocardial injury.

The precise mechanism through which PA induces the expression of URAT1 in cardiomyocytes remains unknown. Matsubayashi^28^ et al. showed that cholesterol metabolite 27-hydroxycholesterol binds to estrogen receptor (ER), and induces SLC22A12 transcription through estrogen response elements (EREs), which results in the upregulation of URAT1 expression in HepG2 cell and the kidney. Furthermore, palmitoylation of ER is essential for ER membrane signaling, which could eventually stimulate EREs.^32^^,^^33^^,^^34^^,^^35^^,^^36^ Considering the possibility that ER also regulates URAT1 expression via EREs in cardiomyocyte, PA taken up into cardiomyocytes through CD36 may bind to ER as well as induce ER palmitoylation, which ultimately increases the URAT1 expression in cardiomyocytes. Further studies are warranted to fully delineate the role of ER in PA-induced upregulation of myocardial URAT1 levels.

A previous study also showed that HFD-induced myocardial injury is partly attributed to the direct effects of PA on cardiomyocytes.^16^ The direct effects of PA on cardiomyocytes involve MAPK phosphorylation, resulting in cellular injury and inflammatory responses,^16^^,^^23^^,^^24^ which is consistent with the findings of the present study. Therefore, the protective effects of dotinurad against PA-induced cardiomyocyte injury are partially due to the inhibition of MAPK phosphorylation. In addition, another previous report showed that p38 and ERK play a critical role in the activation of XO in the lung,^37^ which suggests that PA-induced MAPK phosphorylation also leads to the activation of tissue XO in cardiomyocytes as a pro-oxidant mechanism. In fact, we found that PA overload activates tissue XO activity with a corresponding increase in the UA content in cardiomyocytes, even when cultured in UA-free medium (Figures 6C and 6D). As we and others previously reported,^1^^,^^38^^,^^39^ XO-derived ROS, as well as the enhanced oxidative stress/inflammatory response induced by the excessive accumulation of intracellular UA,^18^^,^^30^ lead to the inhibition of the Cr shuttle, which results in the exacerbation of cardiac energy metabolism through the activation of purine metabolism in association with the acceleration of ATP breakdown. Thus, URAT1-selective inhibition may have a substantial impact on preserving cardiomyocyte energy metabolism in PA-treated cardiomyocytes via the inhibition of MAPK phosphorylation and XO activity. Of note, the present study indicated that these effects occurred via exogenous UA-independent mechanisms.

We found that PA, particularly at high concentrations, substantially reduced cardiomyocyte viability (Figure S7). It is possible that not only apoptosis but also various processes of cell death are involved in PA-induced cellular injury, although further investigations are required to clarify the precise cell death mechanism observed in the present study. Cardiac dysfunction observed in HFD, by contrast, may be attributed to not only reduced cardiomyocyte viability *per se* but also other mechanisms, such as increased cardiac fibrosis (Figures 2B–2D).

In addition to the direct effects of dotinurad on cardiomyocytes, there may be some indirect effects of URAT1-selective inhibition on HFD-induced myocardial injury. Using the same mouse model of diet-induced obesity with dotinurad treatment,^7^ we recently reported that dotinurad treatment ameliorates systemic insulin resistance by attenuating hepatic steatosis and inducing adipose tissue browning in association with the activation of the brown fat thermogenic program. Given that hepatic steatosis produces proinflammatory cytokines such as IL-1β, IL-6, C-reactive protein, and TNF-α, which contribute to endothelial dysfunction and myocardial deformation,^40^ the present findings may also be secondary to the improvement in systemic insulin resistance in association with the inhibition of proinflammatory cytokine production in the liver. Likewise, one cannot exclude the indirect effects of improvement in the kidney function and/or vessels on cardiac health and homeostasis, although at least plasma Cr levels were comparable between HFD and NFD, and dotinurad treatment did not affect them (Figure S2). It is quite possible that PA induces vascular dysfunction (including microvascular dysfunction), while dotinurad improves it, although there were no significant between-group differences, at least in blood pressure (and heart rate), in the present HFD model based on dotinurad treatment, as we previously reported.^7^ However, the possibility remains that modification of the vascular function by URAT1 inhibition may play a significant role in the improvement of the cardiac function in models of diet-induced obesity.

Nonselective URAT1 inhibitors, such as probenecid or benzbromarone, are reported to decrease cardiovascular events in comparison to allopurinol, a classical XO inhibitor.^41^^,^^42^ One of the reasons for the superiority of URAT1 inhibitors in these studies may be the inhibited activity of URAT1 in cardiomyocytes and URAT1-derived cardiomyocyte injury, as indicated in the present study. In addition, URAT1 inhibitors prevented the activity of inflammatory pathways induced by intracellularly transported UA in vascular endothelial and smooth muscle cells, as demonstrated previously.^4^^,^^42^ Taken together, these unique actions of URAT1 inhibitors on the cardiovascular system might be related to the reduction in cardiovascular death, especially in metabolic syndrome. It is worth investigating the effects of the URAT1-selective inhibitor on cardiovascular events in a future clinical study.^43^

In conclusion, URAT1 is expressed in murine heart tissue and cardiomyocytes and actually transports UA into cardiomyocytes. Among various components of metabolic syndrome, PA (but neither insulin + high glucose nor UA *per se*) is a major positive regulator of URAT1 expression, at least in cardiomyocytes, and a primary factor that causes a sequence of pathological findings in the diet-induced metabolic heart. The PA-induced upregulation of URAT1 in cardiomyocytes led to the phosphorylation of MAPK, resulting in the activation of apoptosis, ROS, and inflammation either directly or indirectly through intracellular XO activation in an exogenous UA-independent manner, findings that were ameliorated by treatment with a URAT1-selective inhibitor (Graphical Abstract). These protective roles of the URAT1-selective inhibitor against diet-induced myocardial injury lead to the improvement of cardiac inflammation and fibrosis, which results in the improvement of HFD-induced cardiac dysfunction. Taken together, the results of the present study suggest that URAT1-selective inhibition has therapeutic potential for metabolic heart disease-associated hyperuricemia.

### Limitations of the study

The present study was associated with some limitations. First, the expression of URAT1 was not detected in the glomerulus or collecting ducts in a previous study,^44^ which is inconsistent with the present study. This is partially because the anti-URAT1 antibody used in the present study is different from that used in the previous study. Second, heart tissue contains various types of cells (e.g., cardiomyocytes, vascular smooth muscle cells, vascular endothelial cells, and cardiac fibroblasts), and URAT1 is also expressed in these cells.^4^^,^^5^ The URAT1 expression in cardiomyocytes needs more substantiation by showing its expression in a pure fraction of isolated cardiomyocytes from the current HFD hearts. Furthermore, studies using adult cardiomyocytes isolated from control and HFD-exposed mice could also be used to determine whether or not URAT1 inhibition modifies the remodeling of gene expression and metabolism induced by metabolic stress conditions. In addition, using cell-specific knockdown models of URAT1 *in vivo*, the pathophysiological role of URAT1 can be more specifically explored (including a cardiomyocyte-specific role), which also addresses concerns that the current findings are off-target effects of dotinurad. However, a compensatory mechanism might come into play when URAT1 is knocked out, with other UA transporters, such as URATv1 and MCT9,^45^ receiving greater activation. Indeed, when NRCMs were exposed to UA, particularly at a high dose, dotinurad partially but not completely reduced the UA content, suggesting that other UA transporters might be activated and transport UA into cardiomyocytes (Figure 4B). Thus, it would still be difficult to investigate the URAT1-specific effects on cardiomyocytes even using cardiomyocyte-specific URAT1 knockout models. Third, the precise mechanisms through which PA induces the expression of URAT1 in cardiomyocytes remain unknown as described previously. Further studies are warranted to fully delineate the mechanistic link between PA-induced damage to cardiomyocytes and the modulation of URAT1 activity mediated by dotinurad. In this context, the proposed mechanisms by which URAT1 mediates pathology via inflammation or oxidative stress (including the activation of intracellular XO) remain speculative. Future studies using either overexpression or gain of function of URAT1 systems are warranted to determine whether or not URAT1 actually promotes pathology through inflammation or oxidative stress in cardiomyocytes in diet-induced metabolic heart disease. Also, the extent to which URAT1 inhibition is cardioprotective against other inflammatory stimuli or oxidants could be important investigations in order to further clarify the innate biological characteristics of myocardial URAT1.

## STAR★Methods

### Key resources table

**Table:** 

REAGENT or RESOURCE	SOURCE	IDENTIFIER
**Antibodies**		

Rabbit polyclonal anti-URAT1	Proteintech	14937-1-AP; RRID: AB_2191270
Rabbit monoclonal anti phospho-ERK	Cell Signaling Technology	#4370; RRID: AB_2315112
Rabbit monoclonal anti ERK	Cell Signaling Technology	#4695; RRID: AB_390779
Rabbit monoclonal anti phospho-p38	Cell Signaling Technology	#4511; RRID: AB_2139682
Rabbit monoclonal anti p38	Cell Signaling Technology	#8690; RRID: AB_10999090
Rabbit monoclonal anti phospho-JNK	Cell Signaling Technology	#4668; RRID: AB_823588
Rabbit polyclonal anti JNK	Cell Signaling Technology	#9252; RRID: AB_2250373
Mouse monoclonal anti-β-actin	Sigma Aldrich	A5316; RRID: AB_476743
Rabbit monoclonal anti-NLRP3 antibody	Abcam	ab263899; RRID: AB_2889890
Rabbit polyclonal anti-cleaved-caspase-3	Cell Signaling Technology	#9661; RRID: AB_2341188
Rabbit polyclonal anti-Bax	Cell Signaling Technology	#2772; RRID: AB_10695870
Rabbit polyclonal anti-caspase-3	Cell Signaling Technology	#9662; RRID: AB_331439
Mouse monoclonal anti-caspase-9	Cell Signaling Technology	#9508; RRID: AB_2068620
Rabbit monoclonal anti-GAPDH	Cell Signaling Technology	#2118; RRID: AB_561053
Mouse monoclonal anti-cardiac Troponin T	Thermo Fisher Scientific	MA5-12960; AB_11000742
Goat anti-Mouse IgG1, Alexa Fluor™ 488	Thermo Fisher Scientific	A21121
Goat anti-Mouse IgG(H + L), Alexa Fluor™ 568	Thermo Fisher Scientific	A11011

**Chemicals, peptides, and recombinant proteins**		

Dotinurad	Fuji Yakuhin	N/A
Palmitic acid	Sigma-Aldrich	P0500
Uric acid	Sigma-Aldrich	U2625
Horse Serum	Thermo Fisher Scientific	16050122
TRIzol regent	Thermo Fisher Scientific	15596018
PureLink™ DNase Set	Thermo Fisher Scientific	12185010
High-Capacity cDNA Reverse Transcription Kit	Thermo Fisher Scientific	4368814
TaqMan™ Fast Universal PCR Master Mix (2X)	Thermo Fisher Scientific	4366072
PVDF membrane	Thermo Fisher Scientific	LC2005
Lipofectamine™ RNAiMAX	Thermo Fisher Scientific	13778150

**Critical commercial assays**		

UA assay kit	Sigma-Aldrich	MAK077
Amplex™ Red Xanthine/Xanthine Oxidase Assay Kit	Thermo Fisher Scientific	A22182
LabAssay ™ Creatinine	Fujifilm Wako Pure Chemical Corporation	290–65901
MTS assay	Abcam	ab197010

**Experimental models: Cell lines**		

HUVEC	KURABO	KE-4109

**Experimental models: Organisms/strains**		

C57BL/6NCrSlc	Sankyo Labo Service Corporation, Inc	RRID:MGI:5295404

**Oligonucleotides**		

Silencer Select Negative Control siRNA	Thermo Fisher Scientific	catalog no.4390843
Silencer Select Slc22a12 (URAT1) siRNA	Thermo Fisher Scientific	siRNA ID: s172118

**Software and algorithms**		

GraphPad Prism 8	GraphPad Software	https://www.graphpad.com/
ImageJ	NIH	https://ImageJ.nih.gov/ij/

### Resource availability

#### Lead contact

Further information and requests for resources and regents should be directed to and will be fulfilled by the lead contact, Tomohisa Nagoshi (tnagoshi@jikei.ac.jp).

#### Materials availability

This study did not generate new unique regents

### Experimental model and study participant details

#### Animal models

All animal procedures conformed to the National Institutes of Health Guide for the Care and Use of Laboratory Animals and were approved by the Animal Research Committee at the Jikei University School of Medicine (2021-022, 2021-023). Male C57BL/6 mice at 8 weeks of age were fed either a normal-fat diet (NFD) or a high-fat diet (HFD) for 16–20 weeks as described previously.^7^ HFD (D12492; Research Diets, New Brunswick, NJ, USA) is a lard-based HFD containing 60% kcal fat, 254.5 g/kg fatty acid, 81.5 g/kg saturated fatty acid (SFA), and 51 g/kg palmitic acid (PA). Where indicated, mice fed NFD or HFD received the URAT1-selective inhibitor dotinurad (50 mg/kg/day, kindly provided by Fuji Yakuhin Co., Saitama Japan) for 4 weeks by dietary intake. Four weeks after the initiation of dotinurad treatment, the mice were heparinized (1000 IU/kg, intraperitoneally [i.p.]) and anesthetized (0.3 mg/kg medetomidine, 4.0 mg/kg midazolam, and 5.0 mg/kg butorphanol, i.p.) to eliminate suffering. Then, the heart was excised and washed in PBS (#167–14491, Fujifilm, Wako Pure Chemical Corporation) at 4°C. After washing, tissues were snap-frozen in liquid nitrogen and stored at −80°C until further analysis.

### Method details

#### Plasma PA concentration measurements

Blood was drawn immediately after heart excision and centrifuged (3000 g, 10 min, 4°C). The plasma (supernatant) was collected into new tubes and frozen at −80°C prior to measurement. The plasma PA concentration of the frozen sample was measured by the Japan Institute for the Control of Aging (JaICA), Nikken SEIL CO., LTD (Shizuoka, Japan).

#### Cell culture

Primary cultures of isolated neonatal rat cardiomyocytes (NRCM) were prepared from 1- to 3-day-old Sprague‒Dawley rat heart ventricles using a neonatal cardiomyocyte isolation system (#LK003300, Worthington Biochemical Corp, USA) and plated in 60 mm collagen-coated dishes as previously described.^46^ Cardiomyocytes were cultured in Dulbecco’s modified Eagle’s medium (DMEM) (Gibco) (#11885076, Thermo Fisher Scientific, USA) containing 10% horse serum, 5% fetal bovine serum, 1% penicillin‒streptomycin, and 200 μM bromodeoxyuridine at 37°C in humidified air with 5% CO2.

Cardiac fibroblasts were cultured in Dulbecco’s modified Eagle’s medium (DMEM) (Gibco) (#11885076, Thermo Fisher Scientific, USA) containing 10% horse serum, 5% fetal bovine serum, and 1% penicillin‒streptomycin at 37°C in humidified air with 5% CO2.^47^ The medium was then changed to serum-free DMEM, and the cells were incubated for 12 to 16 h before all experiments. After 1 h or 24 h of stimulation by the indicated treatments (100 or 200 μM PA, 5 or 15 mg/dL UA, 4.5 g/L glucose with or without 100 nM insulin, or 15 μM dotinurad), each dish was snap frozen.

Human vein umbilical vein endothelial cells (HUVECs) were purchased from Kurabo (Osaka, Japan) and grown in HuMedia-EG2 medium (#KE-2150S, Kurabo, Osaka). The medium was then changed to serum-free medium, and the cells were incubated for 12 h before experiments. After 24 h of stimulation by the indicated treatments (100 or 200 μM PA, with or without 15 μM dotinurad), each dish was snap frozen.

#### Preparation of BSA-conjugated PA

Palmitic acid (PA; 128 mg) (#P0500, Sigma Aldrich, Tokyo, Japan) was melted in 1 mL of ethanol at 70°C, and the PA concentration of this solution was 500 mM, as previously described.^48^ Ten microliters of 500 mM PA was added to 1 mL of 10% FFA-free BSA medium (#017–22231, Fujifilm, Wako Pure Chemical Corporation) while being vortexed. PA-BSA was incubated in a heat block at 55°C for 15 min and vortexed, which was repeated twice. The vehicle control was prepared by mixing 10 μL of ethanol with 1 mL of 10% FFA-free BSA medium. The molar ratio of PA-BSA is 3.3:1 (PA:BSA).

#### Determination of cell viability

NRCMs were seeded in 96-well plates and cultured for 6 days until cells became 80% confluent, and treatments (100 or 200 μM PA with or without dotinurad) started. After 24 h of stimulation, cell viability was measured with an MTS cell proliferation assay kit (ab197010, Abcam) with absorbance measured at 495 nm in accordance with the manufacturer’s instructions.^49^

#### RNA isolation, reverse transcription, and real-time PCR (RT-PCR)

Total RNA was extracted from frozen NRCMs, cardiac fibroblasts and frozen heart tissue using TRIzol reagent (Invitrogen), and quantitative real-time PCR was performed using a StepOnePlus Real-time PCR System and the StepOne Software program (Applied Biosystems), as previously described.^7^ The RT‒PCR protocol consisted of one cycle at 95°C for 20 s followed by 40 cycles at 95°C for 1 s and 60°C for 20 s using the primers for URAT1 (Applied Biosystems, Mm01244861_m1 and Rn01479630_g1), TNFα (Applied Biosystems, Mm00443258_m1 and Rn99999017_m1), MCP1 (Applied Biosystems, Mm00441242_m1 and Rn00580555_m1), IL-1β (Applied Biosystems, Mm00434228_m1 and Rn00580432_m1), CD68 (Applied Biosystems, Mm00432403_m1), NLRP3 (Applied Biosystems, Mm00840904_m1) and GAPDH (Applied Biosystems, Mm03302249_g1 and Rn01775763_g1). The transcriptional levels were determined using the ΔΔCt method with normalization to GAPDH.

#### Downregulation of gene expression by siRNA

Small interfering RNAs (siRNA) were obtained from Thermo Fisher Scientific (Silence Select Negative siRNA Control 4390843; Silencer Select URAT1 (Slc22a12) s172118) and transfected into NRCMs using a standard reverse transfection protocol^50^ Briefly, a transfection reagent (Lipofectamine RNAiMAX, Thermo Fisher Scientific) was diluted in OPTI-MEM (Thermo Fisher Scientific) and transfected into NRCMs at a concentration of 10 nM according to the manufacturer’s instructions. Twenty-four hours after transfection, the culture medium was replaced by fresh medium; 24 h later, that is, 48 h after transfection, the medium was then changed to serum-free DMEM, and the cells were incubated for 12 h before all experiments. After 24 h of stimulation by the indicated treatments (100 μM PA, with or without 15 μM dotinurad), each dish was snap frozen and analyzed.

#### Histology and immune staining

The heart was excised, washed in ice-cold PBS, and fixed with 10% formalin. The samples were embedded in paraffin, and 4 mm sections were prepared for histological analyses as described previously.^7^ Hematoxylin and eosin-stained and Masson’s trichrome-stained heart sections were observed using a BZ-X800 microscope (Keyence Corp., Osaka, Japan). The fibrotic area was stained blue, and the normal tissue was stained red. Fibrosis was quantified as the total area ratio of blue staining/tissue region using BZ-X800 Analyzer Software. For immunohistochemical staining, fixed heart and kidney sections were incubated with rabbit polyclonal anti-URAT1 antibody (1:200) (14937-1-AP, Proteintech, Tokyo, Japan, RRID: AB_2191270). The stained images were visualized and captured using a BZ-X800 microscope.

#### Immunofluorescence staining

NRCMs were fixed with 4% paraformaldehyde for 15 min, washed three times with PBS and permeabilized with 0.4% Triton X-100 for 15 min. The cells were then blocked with 0.1% Triton X-100/blocking buffer (#37543, Thermo Fisher Scientific) for 45 min. Subsequently, the cells were incubated at 4°C with anti-cardiac troponin T antibody (1:300, MA5-12960, Thermo Fisher Scientific, AB_11000742) and anti-URAT1 antibody (1:100, 14937-1-AP, Proteintech, Tokyo, Japan, RRID: AB_2191270). The next day, the cells were washed with PBS (3 × 5 min) and incubated with an anti-mouse secondary antibody (Alexa Flour 488 goat IgG1, Thermo Fisher Scientific, A21121, 1:2000, RRID AB_2535764) and anti-rabbit secondary antibody (Alexa Flour 568 goat IgG(H + L), Thermo Fisher Scientific, A11011, 1:2000, RRID AB_143157). The cells were washed with PBS (3 × 5 min) and stained with DAPI (1:1000, D523, DOJINDO, Japan) for 30 min at RT. The specimens were observed using a BZ-X800 microscope (Keyence Corp., Osaka, Japan).

#### Immunoblotting

Immunoblotting was performed as described previously,^7^ with rabbit polyclonal anti-URAT1 (1:1500, 14937-1-AP, Proteintech, Tokyo, Japan, RRID: AB_2191270), rabbit monoclonal anti phospho-ERK (1:2000, #4370, Cell Signaling Technology, RRID: AB_2315112), rabbit monoclonal anti ERK (1:1000, #4695, Cell Signaling Technology, RRID: AB_390779), rabbit monoclonal anti phospho-p38 (1:1000, #4511, Cell Signaling Technology, RRID: AB_2139682), rabbit monoclonal anti p38 (1:1000, #8690, Cell Signaling Technology, RRID: AB_10999090), rabbit monoclonal anti phospho-JNK (1:1000, #4668, Cell Signaling Technology, RRID: AB_823588), rabbit polyclonal anti JNK (1:1000, #9252, Cell Signaling Technology, RRID: AB_2250373), mouse monoclonal anti-β-actin (1:5000, A5316; Sigma Aldrich, Tokyo, Japan, RRID: AB_476743), rabbit monoclonal anti-NLRP3 antibody (1:1000, ab263899, abcam, Tokyo, Japan, RRID: AB_2889890), rabbit polyclonal anti-cleaved-caspase-3; (1:500, #9661, Cell Signaling Technology, Tokyo, Japan, RRID: AB_2341188), rabbit polyclonal anti-Bax (1:1000, #2772, Cell Signaling Technology, Tokyo, Japan, RRID: AB_10695870), rabbit polyclonal anti-caspase-3 (1:1000, #9662, Cell Signaling Technology, Tokyo, Japan, RRID: AB_331439), mouse monoclonal anti-caspase-9; (1:1000, #9508, Cell Signaling Technology, Tokyo, Japan, RRID: AB_2068620), rabbit polyclonal anti-4HNE; (1:1000, ab46545, abcam, Tokyo, Japan, RRID: AB_722490) and rabbit monoclonal anti-GAPDH (1:5000, #2118, Cell Signaling Technology, Tokyo, Japan, RRID: AB_561053). The signals were detected using chemiluminescence.

#### Echocardiography

Echocardiography was performed using a high-resolution Vevo 3100 system (VisualSonics) equipped with a high-frequency ultrasound probe as previously described.^1^ The 2D M-mode was obtained at the level of the papillary muscle and end diastolic thickness of intraventricular septum (IVSd), left ventricular end-diastolic dimension (LVDd), end diastolic thickness of posterior wall (LPWd), and left ventricular end systolic dimension (LVDs) were measured. The left ventricular ejection fraction (LVEF) was calculated using Vevo 3100 software program (VisualSonics). All measurements were obtained in triplicate and averaged.

#### Intracellular UA level of NRCMs

After 60 min of incubation with the indicated concentration of UA (0 mg/dL, 5 mg/dL or 15 mg/dL) with or without dotinurad (15 μM), each dish was washed twice with PBS and snap frozen. NRCMs were collected and lysed in 100 μL of cell lysis buffer (#9803, Cell Signaling Technology, Tokyo, Japan) with PMSF and centrifuged (13,000 g, 10 min, 4°C). The UA level in the supernatant was measured using a UA assay kit (#MAK077, Sigma) according to the manufacturer’s protocol as previously described.^7^ The intracellular UA level of cardiomyocytes was corrected based on the protein concentration of the supernatant measured by the Bradford protein assay.

#### XO activity of NRCMs

NRCMs were collected and lysed in 100 μL M-PER cell protein extraction (#78503, Thermo Fisher Scientific, USA) and centrifuged (14,000 g, 10 min, 4°C). The resulting supernatant was added to a working solution containing Amplex Red reagent (100 μM), xanthine (0.2 mM), and horseradish peroxidase type (0.4 U/ml) and incubated at 37°C for 30 min, and H_2_O_2_ production was measured. Fluorescence readings were made in duplicate in a 96-well plate at Ex/Em = 540/590 nm. The XO activity was corrected by the protein concentration of the supernatant measured by a Bradford assay as previously described.^1^

#### Statistical analysis

All quantitative data are presented as the mean ± standard error of the mean (SEM) and were analyzed using Prism 8 (GraphPad). For the comparison of two datasets, either the Mann‒Whitney U test (nonnormal distribution data) or Student’s *t* test (normal distribution data) was performed. For multiple comparisons among ≧3 groups, one-way ANOVA with Tukey’s method was used for post hoc comparisons. Two-sided p values <0.05 were considered to indicate statistical significance.

## Data Availability

• All data reported in this paper will be shared by the lead contact upon request. • This paper does not report original code. • Any additional information required to reanalyze the data reported in this paper is available from the lead contact upon request.
