# Supplementary material for: URAT1 is expressed in cardiomyocytes and dotinurad attenuates the development of diet-induced metabolic heart disease
Source: iScience. 2023 Aug 25;26(9):107730. doi: 10.1016/j.isci.2023.107730 (PMC10483053; doi:10.1016/j.isci.2023.107730)
Supplement: Document S1. Figures S1–S8 and Tables S1 [file mmc1.pdf]

iScience, Volume *26*

Supplemental information

URAT1 is expressed in cardiomyocytes

and dotinurad attenuates the development

# of diet-induced metabolic heart disease

Yoshiro Tanaka, Tomohisa Nagoshi, Hirotake Takahashi, Yuhei Oi, Rei Yasutake, Akira Yoshii, Haruka Kimura, Yusuke Kashiwagi, Toshikazu D. Tanaka, Masayuki Shimoda, and Michihiro Yoshimura

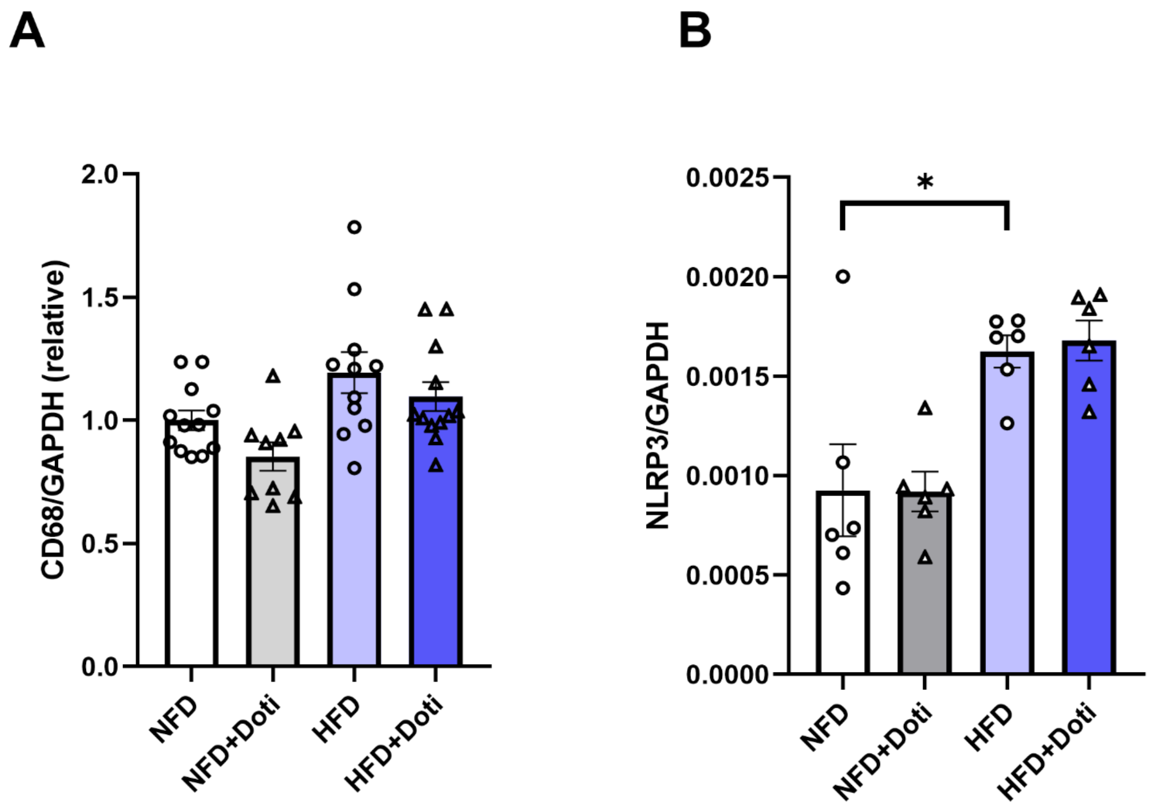

Figure S1. Effects of URAT1-selective inhibitor on CD68 and NLRP3 expressions in the heart, related to Figure 2.

(A and B) CD68 and NLRP3 mRNA expression in either NFD or HFD with or without dotinurad treatment ([CD68] NFD, n=12; NFD+Doti, n=9; HFD, n=11; HFD+Doti, n=12; [NLRP3] n=6 each). Statistical analysis was performed using one-way ANOVA followed with Tukey post-hoc test. *Data are mean* ± SEM. NFD, normal fat diet; HFD, high fat diet; Doti, dotinurad.

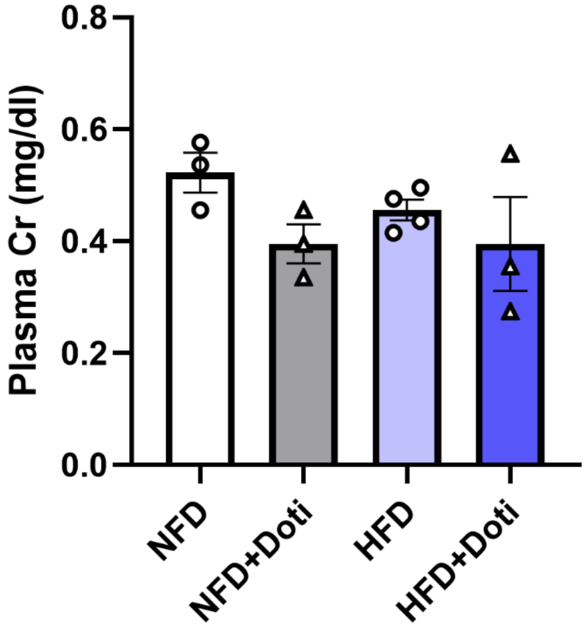

Figure S2. Effects of URAT1-selective inhibitor on plasma creatinine levels, related to Figure 3.

Plasma creatinine levels in either NFD or HFD with or without dotinurad treatment (NFD, n=3; NFD+Doti, n=3; HFD, n=4; HFD+Doti, n=3). *Data are mean* ± SEM. NFD, normal fat diet; HFD, high fat diet; Doti, dotinurad; Cr, creatinine.

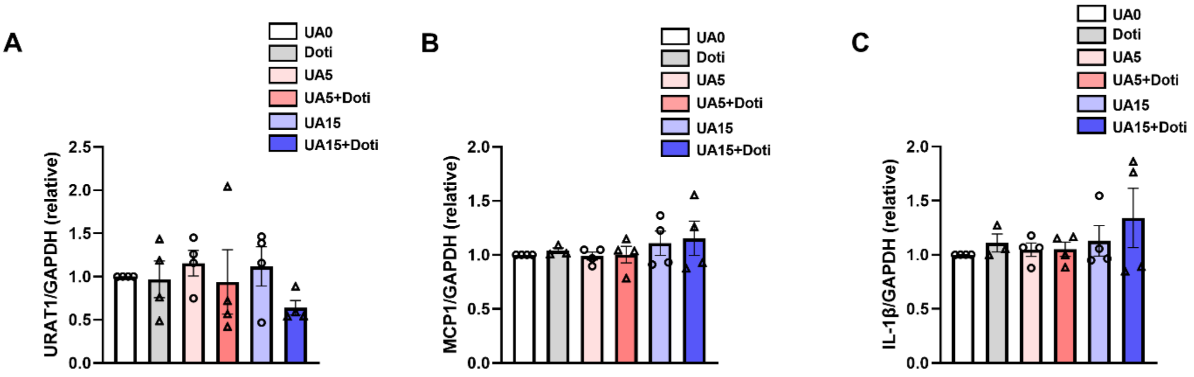

Figure S3. Uric acid did not increase URAT1 expression and inflammatory cytokines in NRCM, related to Figures 5 and 6.

(A-C) URAT1 mRNA, MCP1 mRNA and IL-1β mRNA in NRCM exposed to the indicated treatments for 24 hours ([URAT1] n=4 each; [MCP1] and [IL-1β] UA0, n=4; Doti, n=3; UA5, n=4; UA5+Doti, n=4; UA15, n=4; UA15+Doti, n=4). *Data are mean* ± SEM. UA, uric acid; Doti, dotinurad.

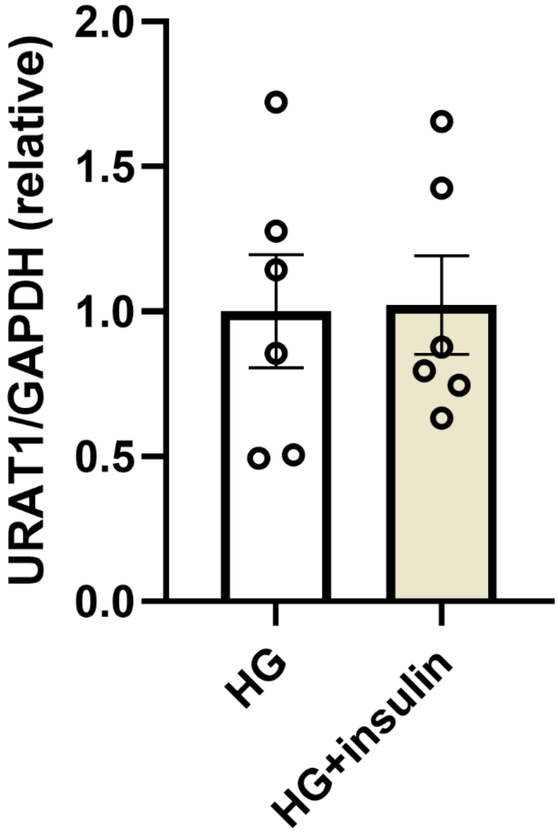

Figure S4. High glucose and insulin did not increase URAT1 expression in NRCM, related to Figure 5.

URAT1 mRNA in NRCM exposed to the indicated treatments for 24 hours (n=6 each). Statistical analysis was performed using two-tailed student t-test. *Data are mean* ± SEM. HG, high glucose.

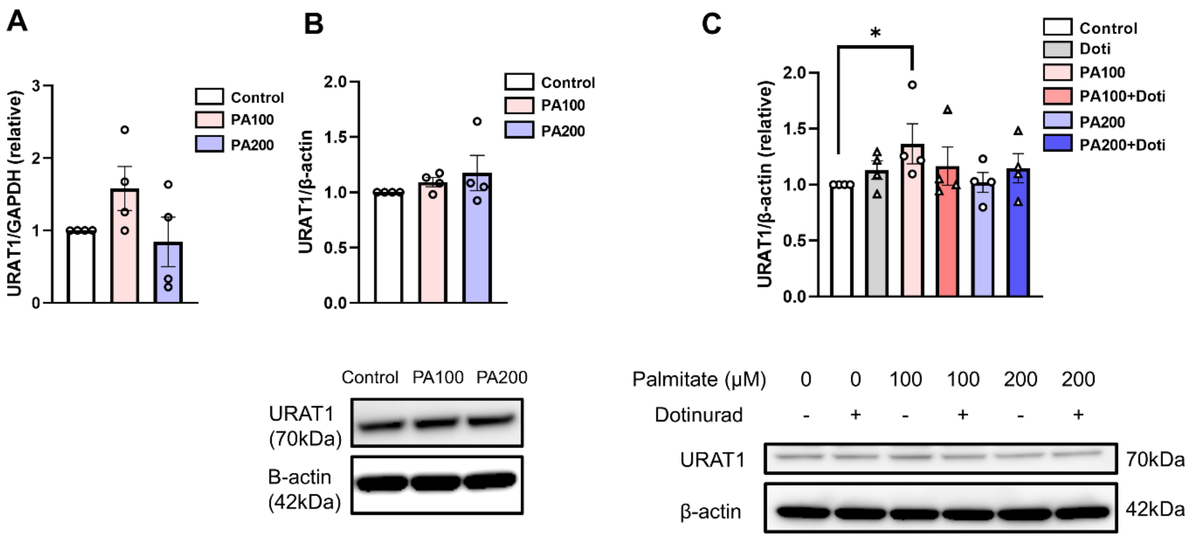

Figure S5. Palmitic acid (PA) did not increase URAT1 expression in cardiac fibroblasts and HUVEC, related to Figure 5.

(A) URAT1 mRNA (n=4 each) in cardiac fibroblast exposed to the indicated treatments for 24 hours. (B) URAT1 protein expressions (n=4 each) in cardiac fibroblast exposed to the indicated treatments for 24 hours. (C) URAT1 protein expressions in HUVEC exposed to indicated treatments for 24 hours (n=4 each). Statistical analysis was performed using Mann-Whitney U test. *Data are mean* ± SEM. *$P<0.05$ between indicated groups. PA, palmitic acid; Doti, dotinurad; HUVEC, human umbilical vein endothelial cells.

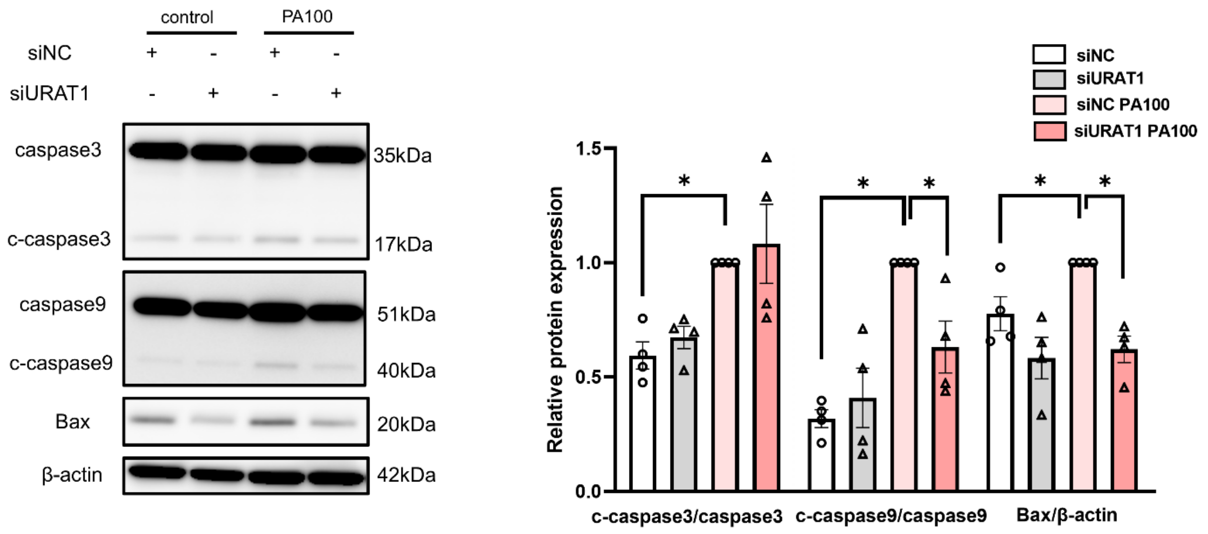

Figure S6. URAT1-knockout partially suppressed PA-induced apoptosis in NRCM, related to Figure 6.

Representative immunoblots (left) and quantitative analysis (right) of c-caspase3, caspase3, c-caspase9, caspase9, Bax and β-actin from NRCM transfected with negative control siRNA (siNC) or URAT1 siRNA (siURAT1) exposed to the indicated treatments for 24 hours (n=4 each). Statistical analysis was performed using Mann-Whitney U test. *Data are mean* ± SEM. *P<0.05 between indicated groups. PA, palmitic acid.

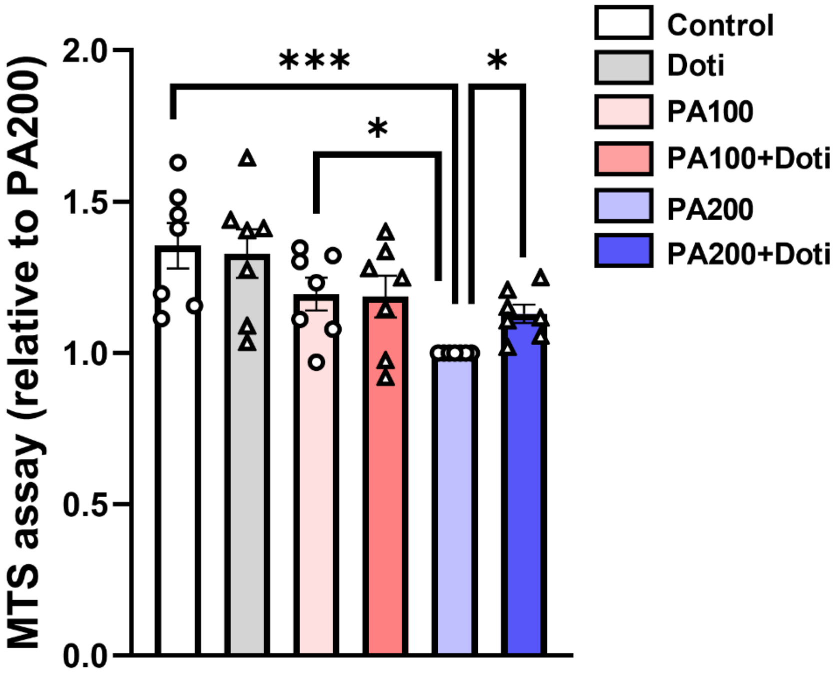

Figure S7. The effects of dotinurad on PA-induced impairment of cellular viability, related to Figure 6.

Effects of dotinurad on NRCM viability after exposure to the indicated treatments for 24 hours (n=7 each). Statistical analysis was performed using Mann-Whitney U test. *Data are mean* ± SEM. ***P<0.001 and *P<0.05 between indicated groups. PA, palmitic acid; Doti, dotinurad.

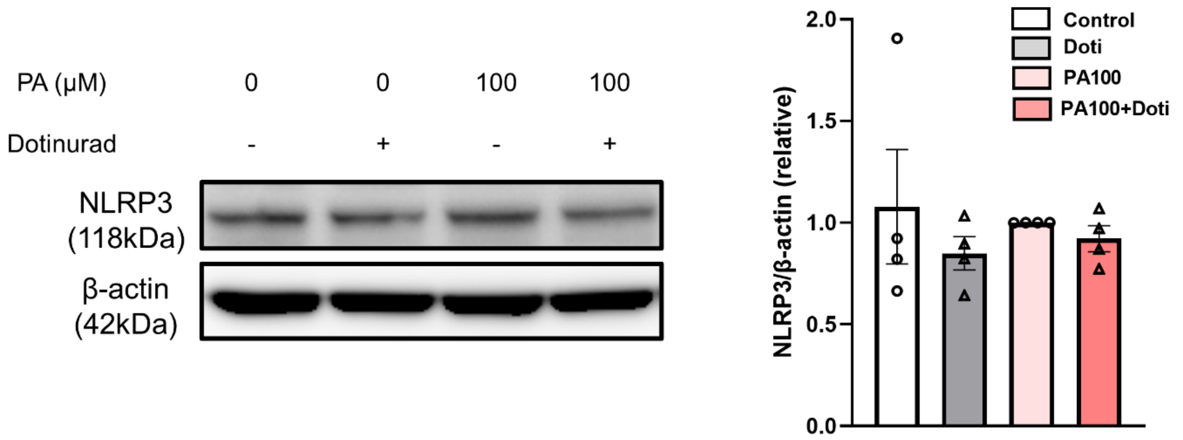

Figure S8. The effects of PA and URAT1-selective inhibitor on NLRP3 in NRCM, related to Figure 6.

NLRP3 mRNA in NRCM exposed to the indicated treatments for 24 hours (n=4 each). Statistical analysis was performed using two-tailed student t-test. *Data are mean* ± SEM. PA, palmitic acid; Doti, dotinurad.

Table S1. Echocardiographic data of NFD and HFD mice treated with or without dotinurad, related to Figure 3.

	NFD (n=11)	NFD+Doti (n=11)	HFD (n=13)	HFD+Doti (n=14)
IVSd, mm	0.73±0.01	0.74±0.02	0.79±0.01*	0.80±0.01
LVDd, mm	3.84±0.09	3.86±0.05	4.21±0.08**	4.12±0.07
LVPWd, mm	0.72±0.01	0.74±0.02	0.80±0.01***	0.79±0.01
LVDs, mm	2.57±0.11	2.54±0.09	3.12±0.11**	2.80±0.06
FS, %	33.3±1.6	34.2±1.7	26.2±1.3**	31.9±1.1†
EF, %	62.4±2.3	63.4±2.4	51.5±2.1**	60.2±1.5††
HR, bpm	551±11	546±10	548±9	546±8

Doti, dotinurad; EF, Ejection fraction; FS, fractional shortening; HR, heart rate; IVSd, interventricular septum diameter; LVDd, left ventricular end diastolic diameter; LVDs, left ventricular end systolic diameter; LVPWd, left ventricular posterior wall thickness at end-diastole.

***P<0.001 versus NFD, *P<0.05 versus NFD, ††P<0.01 versus HFD, †P<0.05 versus HFD. Statistical analyses were performed using one-way ANOVA followed by Tukey's post-hoc test.
